# Facial nerve electrodiagnostics for patients with facial palsy: a clinical practice guideline

**DOI:** 10.1007/s00405-020-05949-1

**Published:** 2020-04-08

**Authors:** Orlando Guntinas-Lichius, Gerd Fabian Volk, Kerry D. Olsen, Antti A. Mäkitie, Carl E. Silver, Mark E. Zafereo, Alessandra Rinaldo, Gregory W. Randolph, Ricard Simo, Ashok R. Shaha, Vincent Vander Poorten, Alfio Ferlito

**Affiliations:** 1 Department of Otorhinolaryngology, Jena University Hospital, Am Klinikum 1, 07747 Jena, Germany; 2 Facial Nerve Center, Jena University Hospital, Jena, Germany; 3 Multidisciplinary Salivary Gland Society, Geneva, Switzerland; 4 Department of Otorhinolaryngology, Mayo Clinic, Rochester, USA; 5 Department of Otorhinolaryngology-Head and Neck Surgery, University of Helsinki and Helsinki University Hospital, Helsinki, Finland; 6 Department of Surgery, University of Arizona College of Medicine, Phoenix, USA; 7 Head and Neck Surgery, MD Anderson Cancer Center, Houston, USA; 8 University of Udine School of Medicine, Udine, Italy; 9 Division of Thyroid and Parathyroid Endocrine Surgery, Department of Otolaryngology, Massachusetts Eye and Ear Infirmary, Boston, USA; 10 Department of Head and Neck Surgery, Guys and St Thomas’ NHS Trust, London, UK; 11 Head and Neck Service, Memorial Sloan-Kettering Cancer Center, New York, NY USA; 12 Otorhinolaryngology-Head and Neck Surgery and Department of Oncology, Section Head and Neck Oncology, University Hospitals Leuven, KU Leuven, Leuven, Belgium; 13 International Head and Neck Scientific Group, Padua, Italy

**Keywords:** Facial nerve, Diagnostics, Facial paralysis, Bell’s palsy, Electroneurography, Electromyography, Electrostimulation, Recommendations

## Abstract

**Purpose:**

Facial nerve electrodiagnostics is a well-established and important tool for decision making in patients with facial nerve diseases. Nevertheless, many otorhinolaryngologist—head and neck surgeons do not routinely use facial nerve electrodiagnostics. This may be due to a current lack of agreement on methodology, interpretation, validity, and clinical application. Electrophysiological analyses of the facial nerve and the mimic muscles can assist in diagnosis, assess the lesion severity, and aid in decision making. With acute facial palsy, it is a valuable tool for predicting recovery.

**Methods:**

This paper presents a guideline prepared by members of the International Head and Neck Scientific Group and of the Multidisciplinary Salivary Gland Society for use in cases of peripheral facial nerve disorders based on a systematic literature search.

**Results:**

Required equipment, practical implementation, and interpretation of the results of facial nerve electrodiagnostics are presented.

**Conclusion:**

The aim of this guideline is to inform all involved parties (i.e. otorhinolaryngologist—head and neck surgeons and other medical specialists, therapeutic professionals and the affected persons) and to provide practical recommendations for the diagnostic use of facial nerve electrodiagnostics.

**Electronic supplementary material:**

The online version of this article (10.1007/s00405-020-05949-1) contains supplementary material, which is available to authorized users.

## Introduction

Guillaume-Benjamin Duchenne in the 1800 s was one of the earliest practitioners of electrodiagnostic [1, 2]. Duchenne noted that patients with persistent facial palsy had absent muscular contractility with nerve stimulation. He, therefore, claimed that electrostimulation could predict prognosis. Blink reflex testing evoked by electrical stimulation was the first neurological test used for facial nerve diagnostics by Kugelberg, in 1952 [3]. In 1963 Laumans and Jongkees introduced the nerve excitability test (NET) [4] for the direct facial nerve and facial muscle testing. With this electrostimulation test, the lowest current eliciting a twitch in the mimetic muscles was defined as the threshold of excitation and the difference in thresholds between the two hemifaces was calculated [5, 6]. May et al. described the maximal stimulation test (MST) [7]. During MST, facial movement of the paretic side was compared with the contralateral side at the stimulation level where the greatest amplitude of facial movement was seen on the normal side. NET and MST both are dependent upon the cooperation of the patient and on the examiner’s visual evaluation of the electrically elicited facial movement. This factor limits the reliability of both tests. These limitations were overcome by electroneurography (ENoG; sometimes also called evoked electromyography). ENoG was popularized by Fisch and Esslen in 1972 [8]. ENoG objectively records the amplitude of electrically evoked muscle action potentials. Finally, electromyography (EMG) measures volitional responses of the facial muscles without electrostimulation. ENoG and EMG are now the most important facial electrodiagnostic tools. Electrostimulation with a visual inspection of the face (as it was used for the NET and MST) still are relevant for facial nerve mapping (FNM). Electrodiagnostics remain valuable tools for patients with facial palsy. Nevertheless, many practitioners do not routinely use them [9]. The situation has been the same for laryngeal electrodiagnostics until recently. To overcome this, the European Laryngological Society published a guideline for laryngeal EMG [10]. The idea behind was to find an agreement on methodology, interpretation, validity, and clinical application of laryngeal EMG as an important step to promote the use of laryngeal electrodiagnostics. The present proposal has now the same goal for facial electrodiagnostics. An overview of the most important facial electrodiagnostic tests is shown in Table 1.

**Table 1:** Overview about the most important facial electrodiagnostic tests

Test	Principle	Comment
Nerve excitability test (NET)	Transcutaneous electrostimulation of the main trunk first on the healthy side, then on the affected side. Examiner watches the patient’s face for the first sign of muscle contraction. Significant side difference of stimulation intensity should indicate poor prognosis	Cannot be recommended as prognostic test due to poor reliability
Maximal stimulation test (MST)	Setup of NET, but supramaximal stimulation. Starts at the main trunk and follows the branching of the facial nerve	Cannot be recommended as prognostic test due to poor reliability A variation of MST with stimulation intensity is used for facial nerve mapping (FNM; see text)
Electroneurography (ENoG)	Setup of NET/MST, increasing stimulation up to supramaximal stimulation, but analysis of the peak-to-peak amplitude of the recorded action potential (CMAP) of the healthy compared to the CMAP of the affected side	Important test between 72 h and 21 days after onset, interpretation of result in comparison to nEMG result (see below)
Needle electromyography (nEMG)	Does not work with external stimulation; The MUAP in the range of the needle electrode is recorded during insertion, at rest, and during voluntary movements	Most important 2–3 weeks after onset of the palsy, because pathologic activity can occur in case of facial nerve degeneration. In the later time course, nEMG is important to detect reinnervation potentials as signs of reinnervation of the facial muscles
Surface electromyography (sEMG)	Like nEMG, sEMG works with voluntary activity of the facial muscles and not with external stimulation. The recording field and therefore the depiction of MUAPs is more superficial but the field is larger than when using nEMG	sEMG is not used for prognostication. Multichannel sEMG is important if the interplay of different facial muscles should be analyzed
Blink reflex	Electrostimulation of the supraorbital branch of the trigeminal nerve (V1) and simultaneous sEMG recording from the orbicularis oculi muscle on both sides	If ENoG and EMG is performed, the additional value is low. Blink reflex testing might be helpful if the lesion site is suspected or lies within the brainstem
Transcranial magnetic stimulation (TMS)	Recording setup like for ENoG, but the stimulation is performed using a magnetic field instead of electric stimulation. Typically, a stimulation over the ipsilateral parietoocciptal region is performed. A stimulation via the contralateral motor cortex is also possible	If ENoG and EMG is performed, the additional value is low for routine cases. TMS is less reliable. It might be helpful in selected cases to confirm an intratemporal lesion site or in unconscious patients

A careful review was done of a comprehensive overview of facial nerve electrophysiology that was published in 2016 [11]. We conducted a systematic literature search for the most recent publications using PubMed and ScienceDirect database, with the following MeSH terms: “facial nerve”, “Bell palsy”, “facial nerve diseases”, “electrodiagnostics”, and “humans” (period: 2015–2019; last search on 21-October 2019) in accordance with the preferred reporting items for systematic reviews and meta-analyses (PRISMA) statement. A total of 32 articles were included in the present review based on relevance and scientific evidence. The highest level of evidence reached the level of retrospective observational cohort studies (Oxford Centre for Evidence-based Medicine Level III–IV). Due to the lack of high-quality evidence, the presented recommendations reached the level of recommendation B, i.e. considerable benefit substantiated by non-first-class evidence, according to international standards and the Association of the Scientific Medical Societies guidelines (Arbeitsgemeinschaft Wissenschaftlich Medizinischer Fachgesellschaften, AWMF; https://www.awmf.org/en/clinical-practice-guidelines/awmf-guidance.html). The most important diagnostics tests are discussed in depth and a consensus is introduced. Recommendations for the use of ENoG and EMG as well as the role of less often employed techniques like blink reflex testing or transcranial magnetic stimulation (TMS) are also presented in detail. The manuscript circulated among the authors in four rounds until a consensus was reached for all recommendations. A strong consensus (Agreement among > 95% of participants) was reached for all recommendations.

These electrophysiological tests are mainly used to determine the severity and prognosis of a peripheral facial nerve lesion. Transcutaneous electrostimulation is helpful for preoperative FNM. EMG in the form of surface EMG (sEMG) is an important tool for facial muscle expression analysis. Intraoperative nerve monitoring was not part of this study: instead, we refer to other recently published recommendations [12, 13]. Facial electrodiagnostics are also used for other diseases than facial nerve palsy i.e. for hemifacial spasm or facial myokymia [14]. Electrodiagnostics for these diseases were not part of this study.

## Facial nerve physiology, pathophysiology, and definitions

The facial nerve can be stimulated volitionally by the first motoneuron in the facial nerve nucleus, or artificially by electrostimulation of the peripheral facial nerve. A sequence of action potentials is then evoked, and the facial nerve is depolarized between the nodes of Ranvier. The action potentials are transmitted to the facial muscles via the motor endplates. A motor unit (MU) consists of a facial motoneuron and its innervated muscle fibers. In small facial muscles, the number of muscle fibers per MU is low. This is needed for the precise motor control of the muscles. The activation of the muscle fibers by its motor neuron results in a motor unit action potential (MUAP). In facial electrodiagnostics, one normally measures the muscle response visually (NET, MST) or through the interpretation of the electrical signals derived from the muscle (ENoG, EMG). Unlike other nerves, direct analysis of only the facial nerve is not possible due to its early extratemporal branching. Most facial nerve injuries take place in the temporal bone. Nerve stimulation proximal to the pathologic lesion site is therefore often not feasible.

Disturbances of facial muscle function can occur from central and peripheral pathology. Peripheral lesions are more relevant for an otorhinolaryngologist—head and neck surgeon. Peripheral facial nerve dysfunction is classified according to Seddon into three subtypes: neurapraxia, axonotmesis, and neurotmesis [15]. Neurapraxia is characterized by functional nerve deficits with no morphological nerve failure. Axonotmesis is defined as a lesion in the axonal nerve fibers, but the covering myelin sheath is intact. Neurotmesis is distinguished as a complete disconnection of the facial nerve. Facial electrodiagnostic testing tries to differentiate between these three aforementioned subtypes.

Depending on the time after a facial nerve injury, electrodiagnostics cannot always differentiate between the three types of nerve injury. The significance of electrodiagnostics can be increased by repeated testing during the time course of a facial nerve lesion. If the lesion lies in the intratemporal segment such as Bell’s palsy, it takes about 72 h before the Wallerian degeneration has reached the extratemporal segment distal to the stylomastoid foramen. Electrostimulation distal to the lesion site such as ENoG can show completely normal results during this 72-h window. The segment will continue to conduct the stimulus as it takes 10–14 days following an intratemporal insult before degeneration reaches the mimetic muscles. Signs of muscle degeneration cannot be seen by EMG before 10–14 days. The final window of post-injury time is at 21 days post-onset. After this time, nerve excitability is definitively lost, and nerve degeneration is complete. Electrodiagnostic studies can be further complicated by the interference of first regenerating fibers, which is why ENoG is most valuable if initially performed 72 h post-onset and repeated several times until day 14–21. After neurotmesis, the degeneration of the muscle takes much longer. EMG can record signs of degeneration up to 3–6 months post-injury.

Synaptic and muscular lesions are typically related to neurological diseases and are relevant mainly for differential diagnosis. Disturbed neuromuscular transmission can be assessed in the facial muscles with diseases such as the ocular form of myasthenia gravis. These patients have a reduced duration of MUAP and an increase of polyphasic potentials in EMG. EMG amplitudes can also be reduced. Furthermore, myopathies can show pathologic spontaneous activity and even a synchronous increase of polyphasic potentials as a sign of simultaneous degeneration and regeneration. Findings of a synaptic or muscular lesion mandate referral of the patient to a neurologist.

## Electrophysiological diagnostic evaluation

The recommended use of facial electrodiagnostics can be found in the clinical guidelines for treating idiopathic facial palsy (Bell’s palsy). The American Academy of Otolaryngology—Head and Neck Surgery Foundation guideline recommends electrodiagnostic testing only for cases of complete paralysis [16]. Patients with incomplete paresis have good prognosis. In contrast, the German and the Spanish guidelines recommend electrodiagnostics for all patients with Bell’s palsy [17–19]. Some recommend electrodiagnostics for all patients with facial nerve disorder. In patients with an invasive malignant parotid tumor, facial nerve infiltration can result in an incomplete paresis when the facial nerve branches peripheral to the main trunk are affected. In these patients, electrodiagnostics can show signs of nerve degeneration. This may be the first suggestion of a malignant lesion and may influence preoperative and surgical planning. Electrodiagnostics can also be helpful to clearly differentiate a central facial palsy from an incomplete peripheral facial palsy that spares the upper face. Patients seen 2–3 months after onset with worsening neurologic findings often have an incomplete facial recovery. In these cases, electrodiagnostic tests provide a clearer picture of the severity of the lesion.

*Recommendation*: Most patients with facial nerve disorder should undergo facial electrodiagnostics.

## Equipment

There are many licensed stationary or portable electrodiagnostic machines that perform all the recommended facial electrodiagnostic tests. The machine should perform ENoG, EMG, and blink-reflex testing. A simultaneous display of at least two EMG channels should also be available. All data should be storable. A bipolar nerve stimulator or adhesive stimulation electrodes are needed for transcutaneous stimulation during ENoG. This stimulator is also needed for transcutaneous FNM. Surface electrodes are needed to record the compound action potential during ENoG and as ground (reference) electrodes. Needle EMG (nEMG) and surface EMG (sEMG) require surface and ground electrode potential differences (in voltage) which are measured during EMG recording. There are monopolar, concentric, bipolar concentric, and single-fiber EMG needle electrodes. The monopolar needle electrode is a stainless-steel needle fully insulated with a thin insulating coating, except for the tip. These solid needles are used for diagnostic purposes. Potentials recorded by monopolar needle electrodes tend to be larger and longer with more phases than those recorded with concentric needle electrodes. For nEMG one uses concentric bipolar needle electrodes. Bipolar electrodes are primarily useful for experimental studies. The same type of electrodes should be used for every routine procedure to facilitate comparison of results in the same patient and between different patients. Solid needle electrodes are needed for facial electrodiagnostics. If an EMG-guided injection, for instance of botulinum toxin is planned, the needle needs to be hollow. For single-fiber EMG (sfEMG), special fine wires are needed for special research purposes. sfEMG is not presented here, as it is not employed for clinical routine facial electrodiagnostics.

*Recommendation*: Facial electrodiagnostic equipment should perform ENoG, and at least 2-channel EMG and blink-reflex testing.

## Electroneurography (ENoG)

**Fig. 1:**
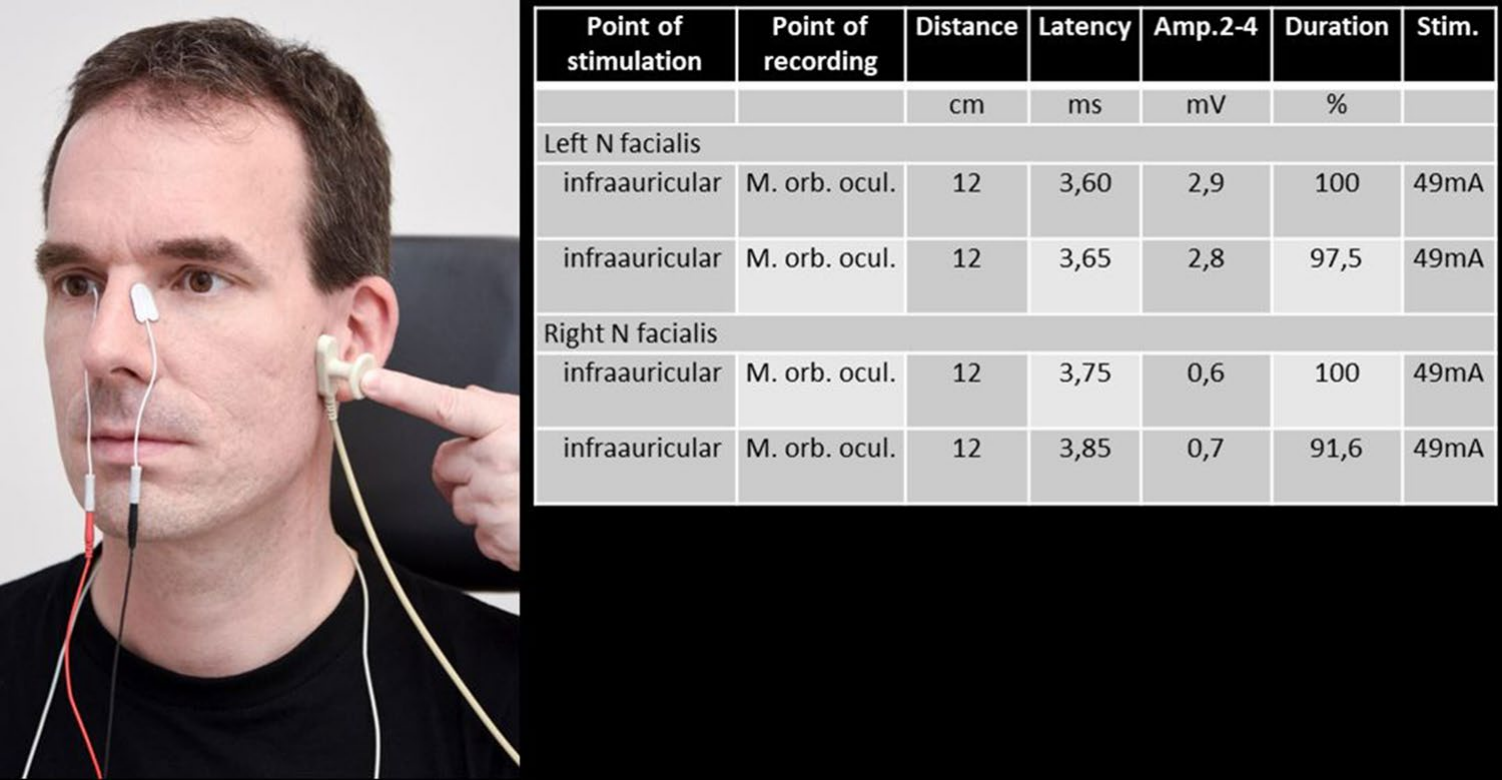
Electroneurography (ENoG) procedure. Recording electrodes placed on each side of the nose. Stimulator placed in front of the ear. Stepwise increased levels of electrical current up to 50 mA. Test results of two repeated measurements of a patient with complete facial paralysis on the right side: Decreased amplitude on the right side with 75–79% amplitude reduction compared to the left side

Electroneurography (ENoG; also called neuronography, electroneuronography, neuromyography, evoked electromyography; Fig. 1) analyzes the evoked compound muscle action potential (CMAP) of a specific facial muscle after transcutaneous stimulation of the main trunk of the facial nerve [8, 20, 21]. The main trunk is stimulated supramaximally at its exit from the stylomastoid foramen with a bipolar stimulator or stimulating electrodes. The CMAP is recorded using a bipolar pair of surface electrodes placed on the target muscle. Typically, ENoG is recorded in the nasolabial fold, the most reliable recording site [22].

ENoG is performed first on the healthy side of the face and then on the affected side. Nerve damage or nerve fiber degeneration leads to a decrease or loss of the CMAP. The amplitude of the CMAP on the affected side is compared to the CMAP of the healthy side and expressed as percent (amplitude of the paralyzed side divided by the amplitude of the normal side). ENoG has a re-test variability of about 20%, [23–25]. The high test–retest variation in ENoG results is the most important criticism of this test [26]. Therefore, it is important to minimize factors (cleaning of the skin, electrode type, room temperature) that can influence results. A side difference of 30% or bigger is considered pathologic. For prognosis, a side difference of 70–95% is considered to be relevant (see below). It is important to note that ENoG alone is not a reliable tool to differentiate between neurapraxia and a more severe lesion [27]. Rarely, ENoG will not show a CMAP, but nEMG still detects voluntary muscle contraction. Fisch called this phenomenon the early de-blocking of the facial nerve fibers [28]. Actually, this finding is still not completely understood.

The lesion site and the onset of an acute palsy are important factors to consider when scheduling and interpreting ENoG. For instance, if the intratemporal portion of the facial nerve is affected (as in Bell’s palsy) it takes about 72 h for Wallerian degeneration to reach the extratemporal nerve distal to the stylomastoid foramen. Since the stylomastoid foramen is the area for electrostimulation for ENoG, ENoG can be normal in the 72 h after onset of such a lesion. Over the next 5–6 days, the response to ENoG will be quickly lost. 21 days after onset, nerve degeneration is complete. ENoG will not change after 21 days. ENoG is helpful for predicting recovery within the time window of 3–21 days [6, 29–32]. The first ENoG is performed at about 3 days after the onset of nerve injury and repeated every 3–5 days [33].

*Recommendation*: ENoG is one of the standard facial electrodiagnostic tests. ENoG is most valuable within the time window of 72 h to 21 days after onset of the lesion. ENoG should be employed and interpreted in combination with EMG.

## Electromyography (EMG)

**Fig. 2:**
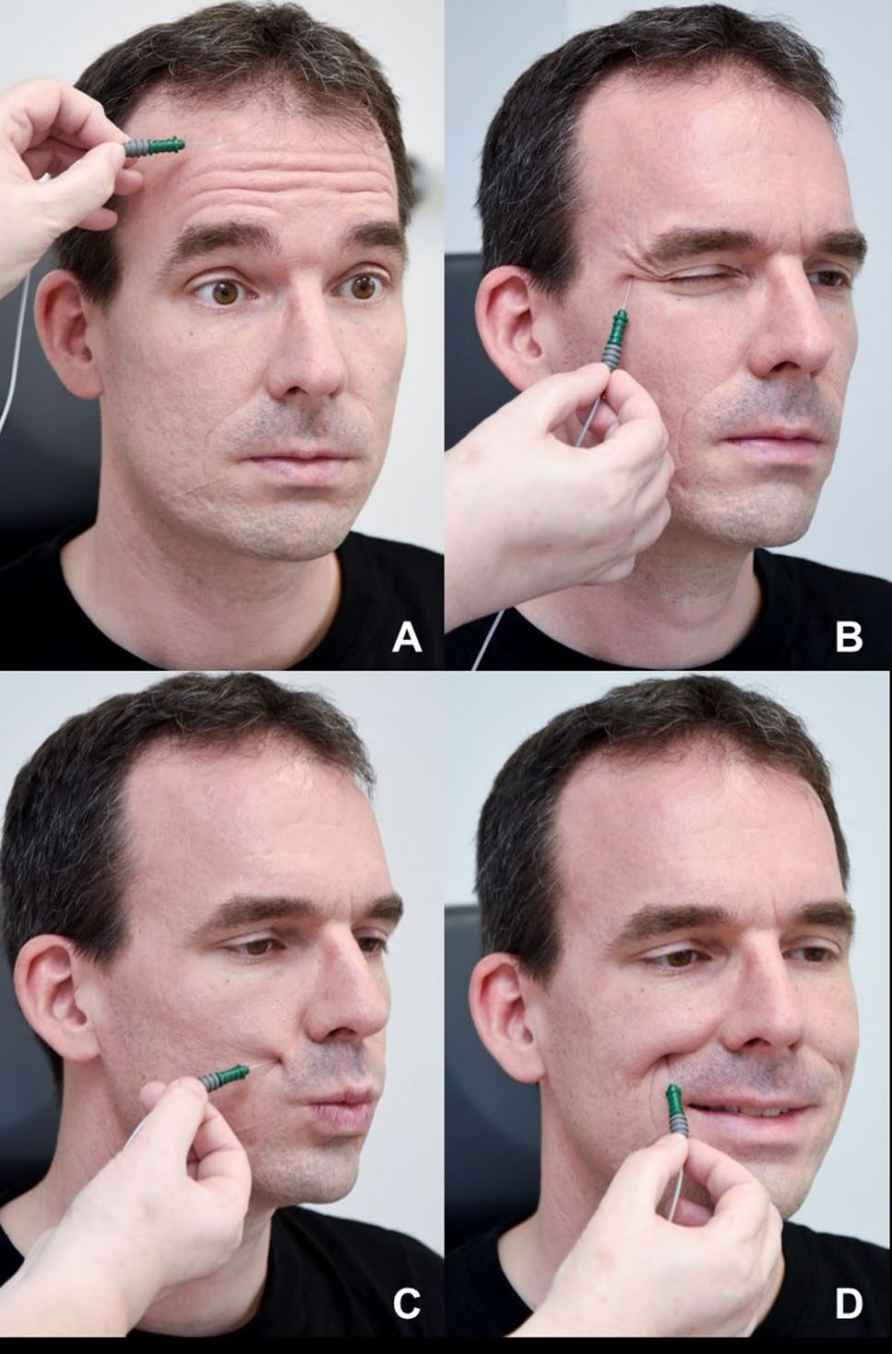
Needle electromyography (nEMG) procedure. **a** frontalis muscle, **b** orbicularis oculi muscle, **c** the orbicularis oris muscle, **d** zygomaticus muscle. nEMG should be performed next to the endplate area of the muscle. nEMG is performed at rest and then during specific tasks

A facial MU consists of a facial motoneuron and all muscle fibers innervated by this motoneuron. Needle EMG (nEMG) is the method used to analyze a facial MUAP recorded from a needle electrode inserted in the facial muscle (Fig. 2). A MUAP can only occur, if a motoneuron action potential activates a muscle. The amplitude and duration of facial MUAP is related to the number of innervated muscle fibers. Amplitude and duration are reduced if the number of innervated muscle fibers are reduced. In contrast to nEMG, surface electromyography (sEMG) is recorded on the skin surface. sEMG records the sum of MUAP in the area of the surface electrode. After the onset of acute facial palsy and before a lesion has become complete, EMG may detect the presence of voluntary MU action potentials. In traumatic cases, this proves that the facial nerve has not been completely transected. This finding directly impacts the decision to explore the lesion site. In contrast to ENoG, EMG is most helpful 2–3 weeks after the onset of the palsy and loss of nerve excitability. Pathological spontaneous activity in the form of fibrillation potentials or positive sharp waves is a sign of facial nerve degeneration. After 4–6 weeks, polyphasic reinnervation potentials are signs of reinnervation of the muscle and can only be seen if facial nerve regeneration occurs.

*Recommendation*: EMG is most valuable in the time frame of 2–3 weeks to 3 months after the onset of a facial nerve injury. EMG is helpful in monitoring for regeneration if reinnervation occurs. EMG should be used and interpreted in combination with clinical examinations.

## Needle EMG (nEMG)

All facial muscles can be studied with one needle type. Two or more needles are needed for the synchronous analysis of several muscles. A reference surface electrode is placed at a myoelectric inactive location of the body such as the area of the manubrium sterni. Bipolar concentric needle electrodes best provide a uniform field for MUAP waveform analysis. Which facial muscles are to be examined depends on what clinical information is needed. If all of the facial nerve is to be investigated, one chooses the frontalis, orbicularis oculi, orbicularis oris, and zygomaticus muscle. These muscles are easy to identify, with mimetic movement.

*Recommendation*: A standardized scheme and sequence should be used for recording facial muscles. An EMG of the frontalis, orbicularis oculi, orbicularis oris, and zygomaticus muscles on the affected side gives a good overview of facial nerve function. If only part of the peripheral facial nerve is affected, nEMG also is abnormal only in the affected area.

nEMG of the muscle of interest should follow a standard recording sequence:

- Insertion activity
- Pathologic spontaneous activity at rest
- Activity during voluntary muscle movement
- Synkinetic activity (in case of chronic palsy)
  After analysis of all muscles of interest, the last steps of nEMG analysis are performed:
- Analysis of the waveform morphology
- Interpretation of the nEMG results

### Insertion activity

In the normal unaffected facial muscle, an insertion activity response occurs during the insertion of the needle into the muscle. The insertion causes bursts of electrical activity for several hundred milliseconds. During early facial nerve injury, the electrical charges surrounding the muscle membrane are unstable. This leads to a prolonged insertion activity. In contrast, the replacement of normal muscle with scar tissue or fat decreases insertion activity.

### Pathologic spontaneous activity at rest

**Fig. 3:**
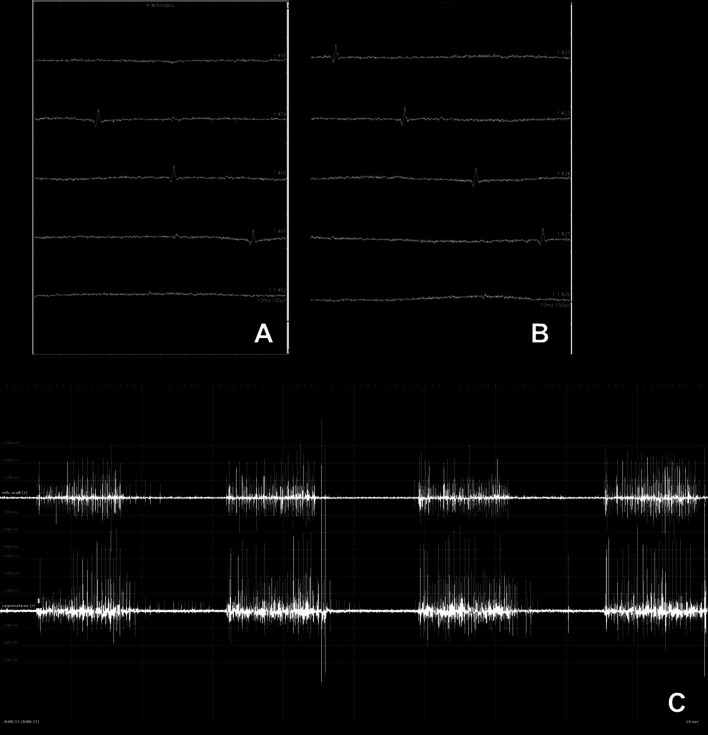
Needle electromyography (nEMG) recordings at rest and during activity: **a**, **b** Two different examples of pathological spontaneous activity as a sign for nerve denervation in patients with facial nerve infiltration by a malignant parotid tumor and facial nerve lesion in temporal bone trauma, respectively, **c** 2-Channel-recording simultaneously of two muscles. Recording of the orbicularis oculi muscle (upper channel) and zygomaticus muscle (lower channel) showing synkinetic activity (setting shown in Fig. 5): While closing the eye not only the orbicularis oculi muscle is activated, but also simultaneously the zygomaticus muscle as a sign for aberrant reinnervation in a patient with post-paretic synkinesis

In the normal unaffected facial muscle, no pathologic spontaneous electrical activity is present at rest. If the patient has problems relaxing the muscle, it is helpful to have the patient tense the muscle before relaxation. When the infratemporal facial nerve fibers are injured, it takes about 10–14 days before degeneration reaches the facial muscle. If the first nEMG is performed early, a repeat measurement should be done at least 14 days later. If the nerve lesion is more distal, degeneration reaches the affected muscles earlier. A denervated facial muscle can show spontaneous activity in the form of unstable electrical charges during the phase of ongoing nerve injury. This pathologic spontaneous activity occurs as fibrillation potentials, complex repetitive discharges, and sharp positive waves (Fig. 3). Most diagnostics are fibrillation potentials. These potentials are characterized as low-amplitude, short-duration units generated by a single muscle fiber. In general, spontaneous activity indicates a poor prognosis for nerve recovery [34]. If nerve regeneration occurs, the muscle receives new electrical impulses and pathologic spontaneous activity decreases and disappears about 2–4 weeks after the nerve injury. When facial nerve damage persists, such as with malignant tumor infiltration, pathologic spontaneous activity can be observed until the nerve is completely destroyed.

### Activity during voluntary muscle movement

**Fig. 4:**
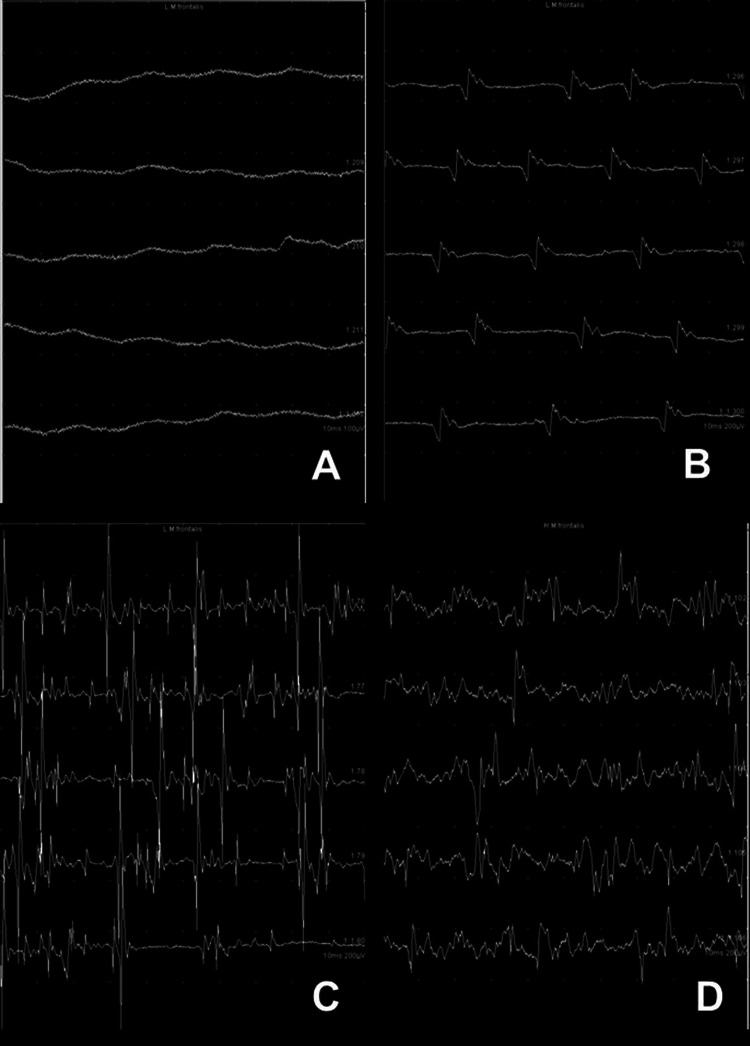
Needle electromyography (nEMG) recordings during voluntary muscle movement showing examples of different activation patterns in the frontalis muscle recorded with a concentric needle electrode 0.45 × 38 mm during contraction: **a** no activity in a patient with acute complete facial paralysis, **b** single-fiber pattern in a patient with acute incomplete facial palsy, **c** decreased recruitment pattern with some polyphasic reinnervation potentials in the phase of regeneration two months after the onset of acute palsy, **d** normal/dense recruitment pattern with some polyphasic reinnervation four months after the onset of acute palsy

This part of the nEMG examination requires the assistance of the patient. The patient is instructed to follow a standard task, frowning for the frontalis muscle, closing the eye for the orbicularis oculi muscle, showing the teeth for the zygomatic muscle etc. The stronger the muscle contraction is, the result is a greater number of activated MU and a denser MU activation pattern (Fig. 4). Facial nerve lesion may lead to a reduced number of normal MUAPs and reduced recruitment during voluntary activity. The patient is instructed to vary the force of activation from minimum to maximum and back. The maximum activation pattern is evaluated. The number of impaired facial nerve axons is directly related to a decrease in the EMG interference pattern. The electrophysiological configuration of MUs is normally not changed in patients with neurapraxia. The duration of MUs and the number of their potential phases typically are increased in patients with axonotmesis or neurotmesis. If the facial nerve regenerates, spontaneously or after nerve repair, facial axons reinnervate the target muscles. A typical sign of reinnervation is polyphasic regeneration potentials. These MUs have greater amplitudes than normal and a prolonged duration during the regeneration process.

### Synkinetic activity

**Fig. 5.**
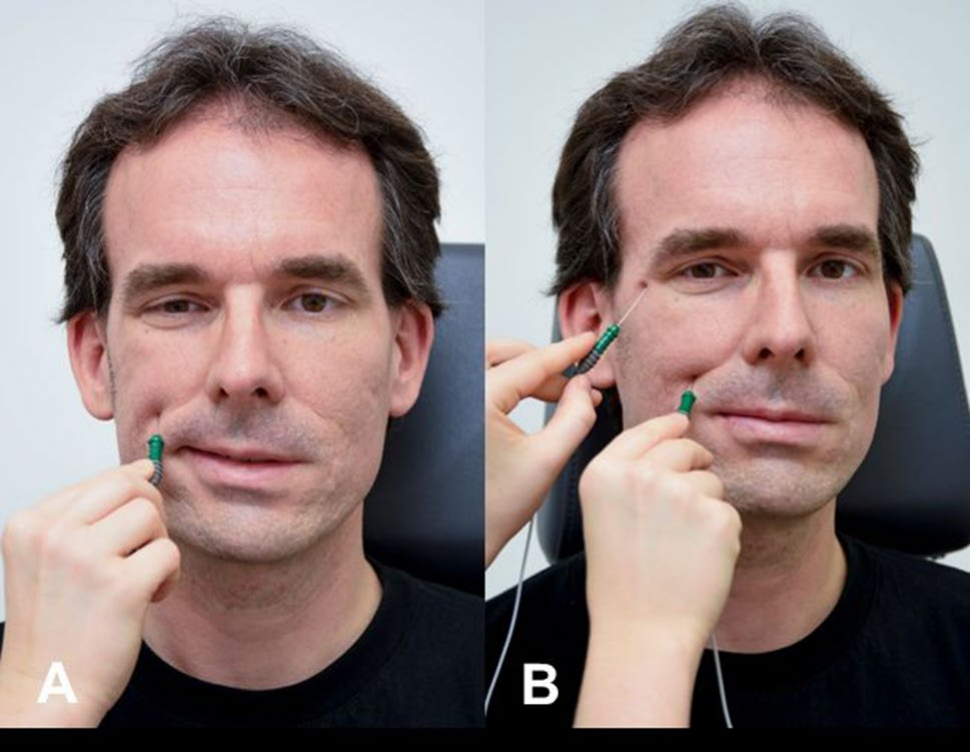
2-channel nEMG setting in patients with post-paretic synkinesis: **a** The simultaneous eye closure is seen during nEMG recording of the zygomatic muscle. **b** To prove the synkinetic activity, it is necessary to perform a simultaneous nEMG of the orbicularis oculi muscle (recording example in Fig. 3)

The regenerating axons branch randomly and reach separate facial muscles. This can finally result in synkinetic activity or post-paretic synkinesis, the involuntary movement of one facial muscle during voluntary movement of another facial muscle. Abnormal nerve regeneration can also lead to more nerve fibers than normal, called hyperinnervation. Clinically, this can cause a strong resting tone and movements, hyperkinesis. During nEMG, the synkinetic activity can be shown by placing the needle electrode in one muscle and having the patient move another facial muscle (Fig. 5). Alternatively, a 2-channel or multiple channel recording is used. Two or more needle electrodes are placed in different muscles. A firing of one MUAP in two or more different muscles is recorded during voluntary contraction and proves synkinetic activity.

### Analysis of the waveform morphology

Facial muscles are smaller than skeletal muscles and therefore, their MUAPs are also smaller. The shape, amplitude, and duration of the MU are the important parts of waveform analysis. A normal facial MU is bi- or triphasic, with a downward positive spike and an upward negative spike. The amplitude is dependent on the needle used. Generally, there is an amplitude of 200–500 mV that lasts about 5–7 ms with muscle-specific normal values. For example, a normal MUAP of the zygomaticus muscle can have 1000–2000 mV. The MUAP amplitude is the result of the number and the strength of the muscle fibers innervated by one axon in a normal facial nerve. In contrast, the duration of the MUAP is determined by the velocity and synchrony of the neural input. MUAPs recorded close to motor end plates can show more than three phases, i.e. polyphasic. There are physiological and normal polyphasic MUAPs that have to be differentiated from polyphasic potentials occurring during regeneration. Normal polyphasic MUAPs will disappear when the needle is shifted away from the endplate region. Due to the high variability of the location of the endplate regions in facial muscles, the interpretation can be more difficult than in big skeletal muscles.

### Interpretation of the nEMG results

The investigator should classify the electrophysiological findings according to neurapraxia, axonotmesis, or neurotmesis [15]. If nEMG results do not show signs of denervation, ENoG, especially when repeated during the facial palsy time course, can be helpful. If the patient with an acute lesion beyond 10–14 days shows normal insertion activity, no pathological spontaneous activity, and a rarified recruitment pattern or single action potentials during voluntary contraction, this is compatible with neurapraxia. If pathological spontaneous activity occurs, axonotmesis should be suspected. A differentiation between axonotmesis and neurotmesis based only on the nEMG is not possible. If the examination detects polyphasic regeneration potentials this indicates reinnervation. If the lesion site is located in the temporal bone or at the main trunk of the peripheral facial nerve, it can take 3–6 months to see the first regeneration potentials [35]. That means that the electrophysiological signs of regeneration precede the clinical signs of recovery. Muscular movement in these situations will not occur until 5–9 months after injury or nerve repair. With recovery there is a continuous increase in the recruitment pattern during voluntary motion. With axonotmesis or neurotmesis, synkinetic activity increases during regeneration and reaches its maximum about 15 months after injury or nerve repair. A specific type of synkinesis is seen in the frontalis muscle. Due to synkinetic reinnervation of antagonistic muscles, the frontalis muscle may not show movement during active frowning; although the nEMG shows muscle activity. This synkinetic phenomenon is called autoparalytic syndrome. One hypothesis for this phenomenon is the simultaneous pathological activation of the frontalis muscle and its antagonist, the orbicularis oculi muscle. In cases of neurotmesis with regeneration, no sign of muscle reinnervation can be detected.

*Recommendation*: nEMG is a helpful tool for monitoring spontaneous facial nerve regeneration or regeneration after facial nerve repair. nEMG can predict future recovery, as reinnervation can be detected about 2–3 months before clinical movements.

## Blink reflex

The physiological involuntary eyelid blink reflex occurs in response to a corneal contact or stimulation by objects that appear rapidly in front of the eye [36–38]. The blink reflex occurs in 0.1 s. The reflex starts via the trigeminal nerve to the trigeminal nucleus, then it descends to the trigeminal spinal nucleus and facial nerve nucleus, from here to the facial nerve’ triggering eyelid closure via the facial nerve. Blink reflex testing takes advantage of the fact that the blink reflex can also be elicited from tactile, electric, optical and auditory stimuli. Since the reflex occurs via the trigeminal nerve (afferent part) to the facial nerve (efferent part), the blink reflex delivers information on facial nerve function with normal trigeminal function. The test can also be used for trigeminal nerve and brainstem testing. The blink reflex is the only clinical test that measures the facial nerve along its entire course. Normally, the amplitude of the repeated stimuli leads to a lower amplitude of the response measured in the orbicularis oculi muscle. Blepharospasm is characterized by involuntary closure of the eyelids and shows a lack of habituation of the blink reflex [39].

Standard blink testing involves electrical stimulation of the supraorbital nerve on the affected side combined with a 2-channel simultaneous sEMG recording from both orbicularis oculi muscles. The exit of the supraorbital nerve in the supraorbital foramen is palpated on the rim of the orbit. Stimulation with 10–20 mA and 0.2 ms duration is used to produce a constant reflex. In routine clinical testing, two responses, R1 and R2, are analyzed. R1 is the fast ipsilateral response of the orbicularis oculi muscle with a latency of about 10–12 ms. The second bilateral response R2 has a latency of about 30–41 ms. R1 has a fixed latency and little variability. R2 is more variable and dependent on arousal and attention. The R2 latency differences between both sides should be less than 5–8 ms [40, 41].

*Recommendation*: If ENoG and nEMG are both performed, the additional benefit of blink reflex testing is low. Blink-reflex testing is a test that allows stimulation of the facial nerve proximal to the lesion site. It may be most helpful if facial nerve damage is suspected to occur within the brainstem.

## Transcranial magnetic stimulation (TMS) and magnetic evoked potentials

Transcranial magnetic stimulation (TMS) uses a magnetic field to stimulate the facial nerve [42]. The responses are recorded from the facial muscles with surface electrodes. Typically, a paranasal recording is used (as for ENoG, cf. Fig. 1). In contrast to ENoG that requires the facial nerve in proximity to a simulated electric field, a magnetic field can pass through the temporal bone and stimulate the facial nerve. TMS of the motor cortex via the cranial bone, is used to elicit a response in the contralateral hemiface. Stimulation in the parieto-occipital region is used to trigger a response in the ipsilateral hemiface. The exact location of the facial nerve stimulation, when stimulating the parieto-occipital region, is unknown.

The ENoG setting with recording and ground electrodes can be used. First, unaffected facial muscles are recorded. Then, the affected side is measured. Like for ENoG, the stimulation intensity is slowly increased. When the amplitude CAMP is not increasing anymore, the intensity is slightly increased for the last time to measure the CAMP with supramaximal stimulation. Typically, a magnetic field of up to 2 T (with a short duration of only 2–3 ms) is generated. The intensity is indicated on the TMS machine in the percentage of the maximal magnetic field output. Normally, about 30–40% of the maximal output is sufficient to obtain supramaximal responses. In contrast to ENoG, one has not to wait 2–5 days before Wallerian degeneration reaches the stimulation site at the stylomastoid foramen because TMS allows a stimulation proximal to the lesion site. Theoretically, TMS should provide information on facial nerve function immediately after the onset of an injury. Unfortunately, TMS did not meet these expectations. Patients with minimally affected nerves also had a complete loss of excitability during TMS [43]. In contrast to ENoG, TMS does not provide the examiner a reliable scalable answer. Consequently, TMS is currently not recommended as a useful electrophysiological test for facial nerve injury. It may be an option for selected cases e.g. unconscious patients to help localize a suspected lesion site in the temporal bone [42, 44].

*Recommendation*: TMS is currently not recommended for routine facial electrodiagnostics. It may prove helpful for highly selected cases of intratemporal pathology.

## Surface electromyography (sEMG)

The coordinated movement of facial muscles is important for facial eye mimic and emotional expression. Facial palsy and other diseases with impaired motor function can lead to impaired mimic and emotional expression. nEMG is used to characterize the degree of and prognostication of nerve damage and is restricted to small muscle areas near the insertion site. Due to its invasiveness, the number of synchronous recordings is limited. nEMG is not an optimal method to monitor muscle function over time. sEMG is the best test to characterize a whole facial muscle and to evaluate inter-muscular coordination. sEMG is not used for prognostication. sEMG is non- invasive, painless, and the number of surface electrodes used and therefore the number of facial muscles analyzed simultaneously, is unlimited. sEMG allows a detailed analysis of the coordinated muscle activation needed for specific tasks and emotional expressions [45, 46]. If several surface electrodes are placed on the same facial muscle, the intramuscular pattern of muscle recruitment can be analyzed. The disadvantage of surface electrodes is that they are not as spatially highly selective as needle electrodes.

sEMG can be recorded with monopolar or bipolar electrodes. The bipolar electrodes are less prone to artificial interference than monopolar recordings. The disadvantage of bipolar electrodes is that they record from superficial portions of the facial muscles. Monopolar electrodes allow reliable whole muscle recording if the positions and inter-electrode distance is held constant to the parallel axis of the muscle fibers. Like nEMG, an indifferent common reference electrode is placed in a muscle-free area. Superficial facial muscles the frontalis, orbicularis oculi, zygomatic, levator labii superioris, levator labii superioris alaeque nasi, orbicularis oris, depressor anguli oris, depressor labii, mentalis muscles are more easily detected with sEMG. If the muscles overlap, such as the depressor anguli oris, depressor labii and mentalis muscles, the sEMG recording will overlap, too.

It is recommended to use small electrodes (for instance, with a diameter of 4 mm). Low-weight cables between electrodes and amplifiers are recommended not to disturb normal muscle movements. Standard movements for grading of facial palsy are used during the sEMG recordings. Each standard movement shows a typical sEMG activity profile. The typical pattern of electrode placement for the recording of specific muscle function are presented in detail elsewhere [47]. The recordings are evaluated offline. Typical parameters for evaluation are mean EMG activity, e.g. the mean rectified EMG amplitude, root mean square, square root of the spectral EMG power, and the analysis for synchronous and sequential activation of certain mimic muscles during the task. Moreover, facial muscle can show different sEMG amplitudes during different tasks. It is also important to notice that an intra-individual side difference of up to 30% is normal. Patients with unilateral facial palsy have sEMG side differences of > 30% between the two sides of the face.

*Recommendation*: Multichannel sEMG is not used for routine facial electrodiagnostics in patients with an acute facial nerve disorder. Multichannel sEMG is recommended only if detailed information on muscle activation with mimic expression is needed or if compensatory movement patterns in patients with chronic facial nerve disorder or post-paralytic synkinesis must be analyzed. This information may be helpful for surgical or medical treatment planning.

## Transcutaneous facial nerve mapping (FNM)

Intraoperative direct facial nerve electrostimulation is widely used to prevent facial nerve damage during surgery. Less often used, although highly reliable, is transcutaneous FNM (Supplement Fig. 1). Park already developed the concept of FNM using transcutaneous electrostimulation in 1998 [48]. Nevertheless, as part of preoperative work-up FNM has so far mainly been established before surgery of vascular malformations [49]. FNM helps to map the course of the peripheral facial nerve and its fine peripheral branches in patients with tumor or scar around the facial nerve [50].

Before surgery, FNM is performed by percutaneous stimulation of the facial nerve and its branches. The resulting facial muscle activations are followed visually. The procedure takes about 25 min. Alternatively, the mapping can also be followed by sEMG and by the recording of the CMAPs. This is much more time-consuming and mainly needed for research questions but not for routine use. A monopolar electrode (for instance, in the form of a ball electrode, 8 mm) is used. A ground electrode is placed. Transcutaneous stimulation with a monopolar ball electrode at the stylomastoid foramen is done at the main trunk of the facial nerve. Electrostimulation is performed with monophasic, rectangular single pulses with a duration of 250 μs. The stimulation at each point starts with 0.1 mA and is increased in 0.1 mA steps. The increased stimulation intensity is stopped when a muscle contraction is seen. When the stimulation at one point is finished, the stimulation electrode is moved forward and the stimulation procedure is repeated. Each point triggering a motor response is marked with a pen on the skin. Ideally, the complete course of the facial nerve and its peripheral branches is marked on the skin at the end of FNM and prior to surgery.

*Recommendation*: FNM can be another tool for surgical planning in complex cases to obtain more information on the general course of peripheral facial nerve branches.

## Documentation of results

Facial electrodiagnostics should be performed and documented in a standardized manner. A recommendation for the testing sequence is shown in Table 2. Standard examinations are ENoG and nEMG. Blink-reflex testing, sEMG, TMS and FNM are reserved for select cases. A data documentation sheet is shown in Table 3.

**Table 2:** Proposal for a routine examination for facial electrodiagnostics

Step	Test	Comment
1.	Prearrangements	Constant conditions are important to reduce re-test variability. Constant room temperature, optimal electrical shielding, regular control of equipment. Abrasive cleaning of the skin areas where the electrodes are to be placed and alcohol cleaning is needed where the stimulator is placed. An adjustable chair/examination couch for the patient is recommended
2.	Electroneurography (ENoG)	Setting example: Sensitivity 10,000 mV; amplifier filtering 1–10 kHz; time frame 10 ms; stimulus duration 200 µs; Maximal stimulus limited to 20 mA; Stimulus rate 1.9 s; Data recorded and averaged using a stimulus rate of 1 Hz with sensitivity adjusted to 2 µV/division and filters set at 30 Hz to 3 kHz a) Ground electrode: Arm or neck; b) Stimulation first on the healthy, then on the paralyzed side; c) Recording electrodes: nasal alae next to each other; d) Stimulator placed on stylomastoid groove; e) Stimulation starts with 0.1 mA and is increased until the maximal CMAP occurs. Stimulation is then once more slightly increased (supramaximal stimulation); f) Storing of the CMAP and measurement of the other side g) Ratio of the peak-to-peak amplitude of the paralyzed side in relation to healthy in percent is calculated
3.	Needle EMG (nEMG)	nEMG of frontalis, orbicularis oculi, oris and zygomaticus muscle on the affected side gives an overview of the facial nerve function. Of course, the selection depends on the facial nerve lesion and the diagnostic questions. The sequence of evaluation is always the same for each muscle: 1. Insertion activity 2. Spontaneous activity at rest 3. Activity during voluntary muscle movement 4. In case of chronic palsy: Synkinetic activity 1. The needle electrode is softly inserted in an oblique angle into the first facial muscle of interest. Normally, the needle is moved during the evaluation to see and hear the optimal placement and recording The muscle activity is graded as follows: a) No activity b) Normal activity (< 300 ms) c) Increased activity d) Highly increased activity 2. The patient is instructed to relax the muscle. The observer should wait a while until the spontaneous activity occurs. Spontaneous activity should be recorded and classified as: a) No reproducible pathologic spontaneous activity b) Little pathologic spontaneous activity c) Moderate pathologic spontaneous activity d) Dense pathologic spontaneous activity 3 The patient is instructed to perform a standard talk for the specific muscle (For instance, frowning for the frontalis muscle, closing the eye for the orbicularis oculi muscle, showing the teeth for the zygomatic muscle). The maximal possible activation of the muscle is documented as follows: a. No activity b. Single fiber pattern c. Severe decreased recruitment pattern d. Mildly decreased recruitment pattern e. Normal/dense recruitment pattern The waveform of the MUAPs is also classified as: a. Normal biphasic motor unit potential b. Early (sometimes polyphasic) reinnervation potentials with low amplitude and long duration c. Giant polyphasic reinnervation potentials with high amplitude and long duration d. Myogenic polyphasic potentials with low amplitude but in many cases normal duration 4. If the patient should be examined for synkinetic activity, step 3 is repeated but the task for another muscle is used, for instance closing the eye while recording from the orbicularis oris muscle. Alternatively, and more precise is to record synchronously an nEMG from different muscles and varying the tasks. Synkinesis is documented as follows: f. Investigated muscles g. Used task h. Few/moderate/strong/very strong synkinesis
Additional test for selected cases		
4.	Blink reflex	Baseline setting like for ENoG or nEMG. Band pass of 20–1000 Hz, pulse duration of 100 µs, repetition rate of 1 Hz, sensitivity of 500 µV/division, and sweep speed of 5 ms/division a) Ground electrode: arm or neck; b) Stimulation normally only the paralyzed side; c) Recording surface electrodes: lateral part of orbicularis oculi muscle on both sides; d) Stimulator placed on supraorbital nerve; e) Stimulation starts with 0.1 mA and is increased until the maximal CMAP occurs. Stimulation is slightly increased (supramaximal stimulation); f) Storing of the CMAP and measurement of the ipsilateral R1 component and of the bilateral R2 component; measurement of the latency of R1 and R2 and of the side difference of the latency of R2. Documentation of the absolute values and interpretation of the results: 1. R1 latency normal (≤ 12 ms) or prolonged (> 12 ms) 2. R2 latency normal (≤ 40 ms) or prolonged (> 40 ms) 3. R2 latency side difference normal (≤ 5 ms) or larger (> 5 ms)
5.	Surface EMG (sEMG)	Baseline setting like for ENoG or nEMG a) Ground electrode: arm or neck; b) Selection of facial muscles and placement of the surface electrodes depends much on the question of the observer; c) Typically, bilateral recordings are performed, but for analysis of synkinetic activity, also unilateral recording may be the best option d) sEMG is recorded while subjects perform facial movements for test purposes, including: pressing the lips together, pulling the corners of the mouth downwards, smiling—pulling the corners of the mouth upwards and backwards, depressing the lower lip, protruding the lower lip, pulling the upper lip upwards, pulling the upper lip upwards and depressing the lower lip simultaneously, pursing lips, blowing out the cheeks, whistling with a similar tone pitch, exhaling forcefully with moderate closed lips (a more diffuse whistling), opening the lips as wide as possible while the jaw is closed, wrinkling the nose, raising the eyebrows up and wrinkling the forehead, contracting the eyebrows, closing the eyelids forcefully, squinting the eyes, closing the right eyelid, closing the left eyelid e) For documentation are important: 1. Analyzed muscles 2. Analyzed tasks 3. Observation of maximal sEMG activity 4. Sequence of recruitment if several facial muscles are involved in the specific task 5. Observation of synchronous and asynchronous activity
6	Transcranial magnetic stimulation (TMS)	Basis setting like for ENoG or nEMG a) Ground electrode: arm or neck; b) Stimulation first on the healthy, then on the paralyzed side; c) Recording electrodes: nasal alae next to each other; d) Magnetic stimulator placed on ipsilateral parieto-occiptal region (in special cases on contralateral motor cortex); e) Typically, a magnetic field of up to 2 T (with short duration of only 2–3 ms) is generated. The intensity is indicated on the TMS machine in percentage of the maximal magnetic field output Stimulation starts with 5% and is increased until the maximal CMAP occurs. Stimulation is slightly increased (supramaximal stimulation); Normally, about 30–40% of the maximal output are sufficient to obtain supramaximal response; f) Storing of the CMAP and measurement of the other side g) Ratio of the peak-to-peak amplitude of the paralyzed side in relation to healthy in percent is calculated
7	Facial nerve mapping (FNM)	FNM is not part of classical facial electrodiagnostics. FNM might be helpful as anpreoperative tool to foresee the course of the peripheral facial nerve and its main branches in the individual patients Baseline setting like for ENoG or nEMG a) Ground electrode: arm or neck; b) Transcutaneous stimulation normally only the paralyzed side; c) Stimulation with monopolar electrode (for instance, all ball electrode, 8 mm); d) Electrostimulation with monophasic, rectangular single pulses with duration of 250 µs. The stimulation at each stimulation point started with 0.1 mA; increase in 0.1 mA steps. Increase of the stimulation intensity is stopped when a muscle contraction is seen; e) Stimulation site is marked with a muscular response is seen at the stimulation place; f) When stimulation at one point is finished, stimulation electrode is moved forward, stimulation procedure is repeated g) Finally, each point triggering a motor response is marked on the skin

**Table 3:** Proposal for documentation of facial ENoG, nEMG, blink reflex testing (adapted from [11])

Name of the patient												
Date of birth	MM-DD-YYYY											
ID												
Diagnosis												
Side of the facial paralysis	R				L				Bilateral	Other		
Comorbidity of relevance for facial electrodiagnostics	Blood thinner? Neurological diseases?											
Date of the examination	MM-DD-YYYY											
Examiner												
Electrodiagnostic equipment used	Of relevance, if there are several workplaces											
ENoG												
Simulation site	Stylomastoid groove/foramen						Other					
Recording site	Nasal alae						Other					
Supramaximal stimulation	mA											
CMAP contralateral side	Side		mV peak-to-peak amplitude									
CMAP affected side	Side		mV peak-to-peak amplitude									
Ratio paralyzed/healthy side	%											
nEMG	Frontalis	Oculi		Oris		Zygomaticus		Other muscle			R	L
Insertion activity												
No activity												
Normal activity (< 300 ms)												
Increased activity												
Highly increased activity												
Pathologic spontaneous activity												
No reproducible pathologic spontaneous activity												
Little pathologic spontaneous activity												
Moderate pathologic spontaneous activity												
Dense pathologic spontaneous activity												
Volitional activity												
No activity												
Single fiber pattern												
Strongly decreased recruitment pattern												
Mildly decreased recruitment pattern												
Normal/dense recruitment pattern												
Morphology of waveform												
Normal biphasic motor unit potential												
Early polyphasic reinnervation potentials with low amplitude and long duration												
Giant polyphasic reinnervation potentials with high amplitude and long duration												
Myogenic polyphasic potentials with low amplitude but normal duration												
Synkinesis	Activity seen in:											
	Frontalis	Oculi		Oris		Zygomaticus		Other muscle			R	L
Task 1: Closing eyes												
Task 2: Pursing lips												
Task 3:												
Task 4:												
Taske5:												
Blink reflex												
Simulation site	Supraorbital nerve						Other					
Recording site	Orbicularis oris muscle, bilateral						Other					
Supramaximal stimulation	mA											
Latency R1 ipsilateral	ms (normal ≤ 12 ms)											
Latency R2 ipsilateral	ms (normal ≤ 40 ms)											
Latency R2 contralateral	ms (normal ≤ 40 ms											
Latency R2 side difference	ms (normal ≤ 5 ms)											
Interpretation												

*Recommendation*: Documentation and evaluation of results should be recorded on a standardized form.

## Conclusion

Otorhinolaryngologists and head and neck surgeons should have basic knowledge of facial electrodiagnostics. ENoG and nEMG are facial electrodiagnostic tests most helpful for investigating patients with acute peripheral facial palsy. Validity is increased when the studies are repeated during the acute phase of the disease. nEMG is an important electrodiagnostic tool to monitor spontaneous regeneration in these patients or regeneration after facial nerve reconstruction surgery. Blink-reflex testing is only of additive value in selected cases. Multi-channel sEMG is a good tool to analyze mimic and emotional expressions in patients with post-paretic synkinesis. Transcutaneous FNM can define preoperatively the topographic cutaneous course of the peripheral facial nerve and its branches in complex surgical cases.

## Electronic supplementary material

Below is the link to the electronic supplementary material.

Supplementary file1 (PDF 84 kb)

## References

[1] Adour KK, Jackler RK, Brackmann DE (1994). Facial nerve electrical testing. Neurotology.

[2] Schaitkin BM, May M, Klein SR, May M, Schaitkin BM (2000). Topognostic, otovestibular, and electrical testing: diagnosis and prognosis. The facial nerve.

[3] Kugelberg E (1952). Facial reflexes. Brain Cogn.

[4] Laumans EP, Jongkees LB (1963). On the prognosis of peripheral facial paralysis of endotemporal origin. Ann Otol Rhinol Laryngol.

[5] Ozgur A, Semai B, Hidir UU, Mehmet Fatih O, Tayfun K, Zeki O (2010). Which electrophysiological measure is appropriate in predicting prognosis of facial paralysis?. Clin Neurol Neurosurg.

[6] Lee DH (2016). Clinical efficacy of electroneurography in acute facial paralysis. J Audiol Otol.

[7] May M, Harvey JE, Marovitz WF, Stroud M (1971). The prognostic accuracy of the maximal stimulation test compared with that of the nerve excitability test in bell’s palsy. Laryngoscope.

[8] Fisch U, Esslen E (1972). Total intratemporal exposure of the facial nerve. Pathologic findings in bell’s palsy. Arch Otolaryngol.

[9] Plumbaum K, Volk GF, Boeger D, Buentzel J, Esser D, Steinbrecher A, Hoffmann K, Jecker P, Mueller A, Radtke G, Witte OW, Guntinas-Lichius O (2017). Inpatient treatment of patients with acute idiopathic peripheral facial palsy: a population-based healthcare research study. Clin Otolaryngol.

[10] Volk GF, Hagen R, Pototschnig C, Friedrich G, Nawka T, Arens C, Mueller A, Foerster G, Finkensieper M, Lang-Roth R, Sittel C, Storck C, Grosheva M, Kotby MN, Klingner CM, Guntinas-Lichius O (2012). Laryngeal electromyography: a proposal for guidelines of the European Laryngological Society. Eur Arch Otorhinolaryngol.

[11] Scholle HC, Schumann NP, Volk GF, Guntinas-Lichius O, Schaitkin B (2016). Electrophysiology. Facial nerve disorders and diseases: diagnosis and management.

[12] Thielker J, Grosheva M, Ihrler S, Wittig A, Guntinas-Lichius O (2018). Contemporary management of benign and malignant parotid tumors. Front Surg.

[13] Guntinas-Lichius O, Silver CE, Thielker J, Bernal-Sprekelsen M, Bradford CR, De Bree R, Kowalski LP, Olsen KD, Quer M, Rinaldo A, Rodrigo JR, Sanabria A, Shaha AR, Takes RP, Vander Poorten V, Zbaren P, Ferlito A (2018). Management of the facial nerve in parotid cancer: preservation or resection and reconstruction. Eur Arch Otorhinolaryngol.

[14] Oge AE, Yayla V, Demir GA, Eraksoy M (2005). Excitability of facial nucleus and related brain-stem reflexes in hemifacial spasm, post-facial palsy synkinesis, and facial myokymia. Clin Neurophysiol.

[15] Seddon H (1943). Three types of nerve injury. Brain.

[16] Baugh RF, Basura GJ, Ishii LE, Schwartz SR, Drumheller CM, Burkholder R, Deckard NA, Dawson C, Driscoll C, Gillespie MB, Gurgel RK, Halperin J, Khalid AN, Kumar KA, Micco A, Munsell D, Rosenbaum S, Vaughan W (2013). Clinical practice guideline: Bell’s palsy. Otolaryngol Head Neck Surg.

[17] Heckmann JG, Lang C, Glocker FX, Urban P, Bischoff C, Weder B, Reiter G, Meier U, Guntinas-Lichius O (2012). The new s2k awmf guideline for the treatment of bell’s palsy in commented short form. Laryngorhinootol.

[18] Lassaletta L, Morales-Puebla JM, Altuna X, Arbizu A, Aristegui M, Batuecas A, Cenjor C, Espinosa-Sanchez JM, Garcia-Iza L, Garcia-Raya P, Gonzalez-Otero T, Manos M, Martin C, Moraleda S, Roda JM, Santiago S, Benitez J, Cavalle L, Correia V, Estevez JM, Gomez J, Gonzalez R, Jimenez J, Lacosta JL, Lavilla MJ, Penarrocha J, Polo R, Garcia-Purrinos F, Ramos F, Tomas M, Uzcanga M, Vallejo LA, Gavilan J (2019). Facial paralysis: Clinical practice guideline of the Spanish society of otolaryngology. Acta Otorrinolaringol Esp.

[19] Heckmann JG, Urban PP, Pitz S, Guntinas-Lichius O, Gagyor I (2019). The diagnosis and treatment of idiopathic facial paresis (bell's palsy). Dtsch Arztebl Int.

[20] Esslen E (1977). The acute facial palsies: Investigations on the localization and pathogenesis of meato-labyrinthine facial palsies. Schriftenr Neurol.

[21] Fisch U (1980). Maximal nerve excitability testing vs electroneuronography. Arch Otolaryngol.

[22] Kim SH, Ryu EW, Yang CW, Yeo SG, Park MS, Byun JY (2016). The prognostic value of electroneurography of bell’s palsy at the orbicularis oculi versus nasolabial fold. Laryngoscope.

[23] Neuwirth-Riedl K, Burian M, Nekahm D, Gstottner W (1990). Optimizing of electroneuronography of the facial nerve. ORL J Otorhinolaryngol Relat Spec.

[24] Coker NJ (1992). Facial electroneurography: analysis of techniques and correlation with degenerating motoneurons. Laryngoscope.

[25] Sittel C, Guntinas-Lichius O, Streppel M, Stennert E (1998). Variability of repeated facial nerve electroneurography in healthy subjects. Laryngoscope.

[26] Adour KK, Sheldon MI, Kahn ZM (1980). Maximal nerve excitability testing versus neuromyography: prognostic value in patients with facial paralysis. Laryngoscope.

[27] Grosheva M, Wittekindt C, Guntinas-Lichius O (2008). Prognostic value of electroneurography and electromyography in facial palsy. Laryngoscope.

[28] Fisch U (1984). Prognostic value of electrical tests in acute facial paralysis. Am J Otol.

[29] Takemoto N, Horii A, Sakata Y, Inohara H (2011). Prognostic factors of peripheral facial palsy: multivariate analysis followed by receiver operating characteristic and Kaplan-Meier analyses. Otol Neurotol.

[30] Byun H, Cho YS, Jang JY, Chung KW, Hwang S, Chung WH, Hong SH (2013). Value of electroneurography as a prognostic indicator for recovery in acute severe inflammatory facial paralysis: a prospective study of Bell's palsy and Ramsay Hunt syndrome. Laryngoscope.

[31] Volk GF, Klingner C, Finkensieper M, Witte OW, Guntinas-Lichius O (2013). Prognostication of recovery time after acute peripheral facial palsy: a prospective cohort study. BMJ Open.

[32] Arslan HH, Satar B, Yildizoglu U, Edizer DT, Akgun H (2014). Validity of late-term electroneurography in Bell’s palsy. Otol Neurotol.

[33] Remenschneider AK, Michalak S, Kozin ED, Barber S, De Venecia RK, Hadlock TA, Jung DH (2017). Is serial electroneuronography indicated following temporal bone trauma?. Otol Neurotol.

[34] Grosheva M, Guntinas-Lichius O (2007). Significance of electromyography to predict and evaluate facial function outcome after acute peripheral facial palsy. Eur Arch Otolaryngol.

[35] Flasar J, Volk GF, Granitzka T, Geissler K, Irintchev A, Lehmann T, Guntinas-Lichius O (2017). Quantitative facial electromyography monitoring after hypoglossal-facial jump nerve suture. Laryngoscope Investig Otolaryngol.

[36] Esteban A (1999). A neurophysiological approach to brainstem reflexes. Blink reflex Neurophysiol Clin.

[37] Pearce JM (2008). Observations on the blink reflex. Eur Neurol.

[38] Kimura J, Powers JM, Van Allen MW (1969). Reflex response of orbicularis oculi muscle to supraorbital nerve stimulation. Study in normal subjects and in peripheral facial paresis. Arch Neurol.

[39] Benbir G, Kiziltan ME (2014). Blink reflex studies in postparalytic facial syndrome and blepharospasm: trigeminal and extratrigeminal somatosensory stimulation. J Clin Neurophysiol.

[40] Kennelly KD (2012). Electrodiagnostic approach to cranial neuropathies. Neurol Clin.

[41] Muzyka IM, Estephan B (2018). Electrophysiology of cranial nerve testing: trigeminal and facial nerves. J Clin Neurophysiol.

[42] Happe S, Bunten S (2012). Electrical and transcranial magnetic stimulation of the facial nerve: diagnostic relevance in acute isolated facial nerve palsy. Eur Neurol.

[43] Schriefer TN, Mills KR, Murray NM, Hess CW (1988). Evaluation of proximal facial nerve conduction by transcranial magnetic stimulation. J Neurol Neurosurg Psychiatr.

[44] Hur DM, Kim SH, Lee YH, Kim SH, Park JM, Kim JH, Yong SY, Shinn JM, Oh KJ (2013). Comparison of transcranial magnetic stimulation and electroneuronography between Bell’s palsy and Ramsay Hunt syndrome in their acute stages. Ann Rehabil Med.

[45] Frigerio A, Cavallari P, Frigeni M, Pedrocchi A, Sarasola A, Ferrante S (2014). Surface electromyographic mapping of the orbicularis oculi muscle for real-time blink detection. JAMA Facial Plast Surg.

[46] De Letter M, Vanhoutte S, Aerts A, Santens P, Vermeersch H, Roche N, Stillaert F, Blondeel P, Van Lierde K (2017). Facial nerve regeneration after facial allotransplantation: a longitudinal clinical and electromyographic follow-up of lip movements during speech. J Plast Reconstr Aesth Surg.

[47] Schumann NP, Bongers K, Guntinas-Lichius O, Scholle HC (2010). Facial muscle activation patterns in healthy male humans: a multi-channel surface emg study. J Neurosci Meth.

[48] Park JI (1998). Preoperative percutaneous facial nerve mapping. Plast Reconstr Surg.

[49] Bly RA, Holdefer RN, Slimp J, Kinney GA, Martinez V, Manning SC, Perkins JA (2018). Preoperative facial nerve mapping to plan and guide pediatric facial vascular anomaly resection. JAMA Otolaryngol Head Neck Surg.

[50] Raslan A, Guntinas-Lichius O, Volk GF (2019). Altered facial muscle innervation pattern in patients with postparetic facial synkinesis. Laryngoscope.

