# Supplementary material for: Facial nerve electrodiagnostics for patients with facial palsy: a clinical practice guideline
Source: Eur Arch Otorhinolaryngol. 2020 Apr 8;277(7):1855–74. doi: 10.1007/s00405-020-05949-1 (PMC7286870; doi:10.1007/s00405-020-05949-1)
Supplement: Supplementary file 1 — Supplementary file1 (PDF 84 kb) [file 405_2020_5949_MOESM1_ESM.pdf]

Supplement Figure 1

Guntinas-Lichius et al.

Facial nerve electrodiagnostics for patients with facial palsy - a clinical practice guideline

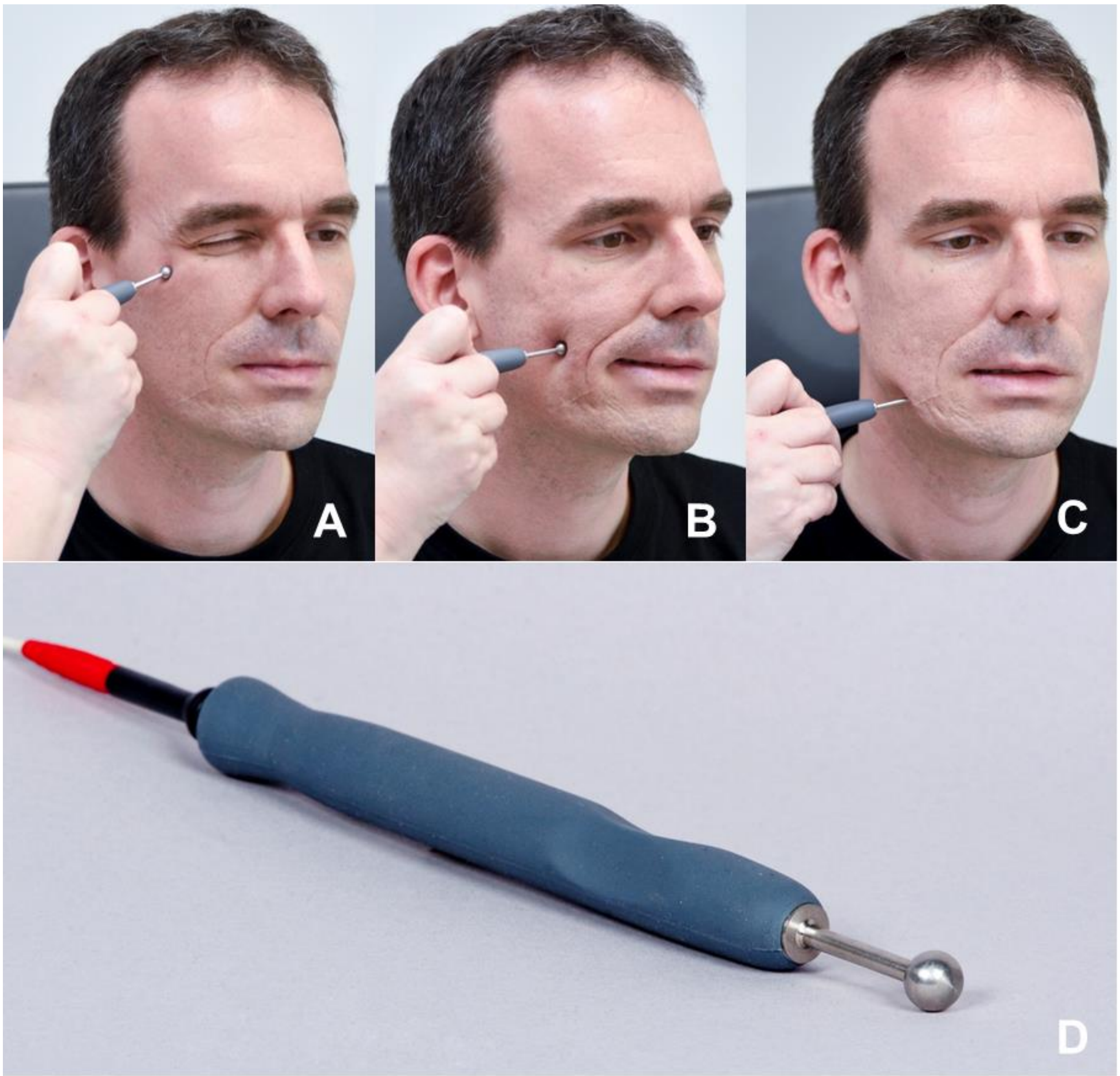

Supplement Figure 1. Facial nerve mapping (FNM). **A**: FNM of a zygomatic branch innervating the orbicularis oculi muscle; **B**: of a buccalis branch innervating the zygomaticus muscle; **C**: of a marginalis branch innervating the depressor anguli oris. **D**: Example of a ball electrode used as stimulator during FNM.
